# Autophagy tunes chondrocyte differentiation and joint developmental precision in zebrafish

**DOI:** 10.1080/27694127.2022.2071189

**Published:** 2022-05-03

**Authors:** Joanna J. Moss, Martina Wirth, Sharon Tooze, Chrissy L. Hammond, Jon D. Lane

**Affiliations:** a School of Biochemistry, University of Bristol, Bristol, UK; b School of Physiology, Pharmacology and Neuroscience, University of Bristol, Bristol, UK; c Molecular Cell Biology of Autophagy, The Francis Crick Institute, London, UK

**Keywords:** ATG13, cartilage, chondrocyte, osteoarthritis, zebrafish

## Abstract

Cartilage is a crucial component of the developing and functioning skeleton. It establishes a template for bone formation and comprises the articular cartilage for smooth joint movement. It is formed by dedicated matrix-secreting cells (chondrocytes) whose development and survival are negatively affected by disorders of crucial homeostatic and metabolic pathways, and by impaired ER and/or mitochondrial stress responses. As these processes are directly influenced by macroautophagy/autophagy, dysregulation of autophagy control in chondrocytes and their progenitors can contribute to human skeletal disorders, notably osteoarthritis (OA). To understand more about the contributions of autophagy during chondrogenesis, we characterized jaw joint development in a new zebrafish *atg13* knockout line with reduced autophagic flux. In this model, embryonic lethality associated with restricted mouth opening range and premature chondrocyte hypertrophy are observed. Our data suggest that autophagy is required for timely chondrocyte maturation and extracellular matrix deposition, findings that highlight the importance of autophagy during normal joint formation.

Osteoarthritis (OA) is a degenerative joint disorder and is one of the biggest causes of disability, making it an increasing socioeconomic concern in countries with an aging population. It is characterized by progressive cartilage degeneration that can affect all major joint structures, causing synovial inflammation and osteophyte (bone spur) formation, accompanied by ligament damage, bone misalignment, and joint pain. Recent studies have correlated inaccurate joint shaping during skeletogenesis with elevated risk of OA in later life. This highlights the need for a clearer understanding of the mechanisms coordinating cartilage and joint development—including how key cellular pathways such as autophagy shape these processes—for a better appreciation of the pathogenesis of OA, and to identify potential targets for its prevention and/or treatment.

Chondrogenesis starts with the condensation and differentiation of mesenchymal stem cells into chondrocytes that delineate the location of future skeletal deployment. Chondrocytes secrete a characteristic extracellular matrix formed largely of COL2A1 (collagen type II alpha 1 chain), along with proteoglycans including ACAN (aggrecan). As they develop and mature, chondrocytes progress through stages of intercalation and proliferation, flattening and separating to form a single-cell stacked column of disc-shaped cells. These undergo hypertrophication as they exit the cell cycle and switch to secreting COL10A1 (Figure 1). Importantly, positive correlations exist between autophagy activity and chondrocyte proliferation and differentiation during early stages of chondrogenesis. Indeed, autophagy is induced in maturing mouse chondrocytes, and when disrupted, growth plate activity is suppressed, leading to growth retardation and premature OA onset.

**Figure 1.**
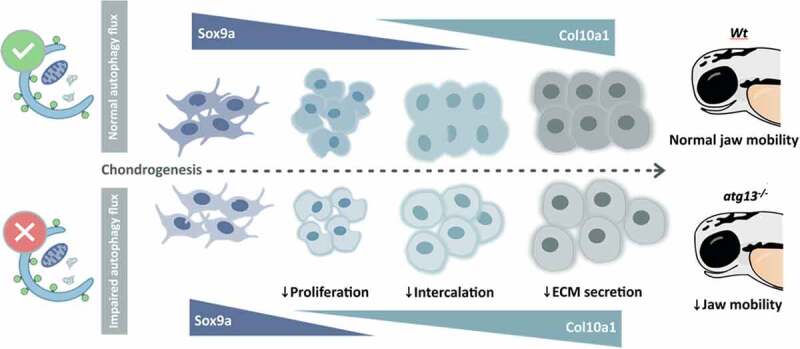
Autophagy is required for timely chondrocyte maturation and proper jaw function in zebrafish larvae. Impaired autophagy flux in *atg13* mutant chondrocytes causes a reduction in Sox9a expression and increased Col10A1 expression compared to *Wt*, indicative of premature chondrocyte hypertrophy, and a disruption to chondrocyte intercalation, resulting in reduced jaw mobility in 5 dpf larvae.

Zebrafish provide an excellent model system to study joint formation, function, and decline, as their skeletal physiology is comparable with mammals, sharing the same joint types and components such as joint cavities, articular cartilage, and synovial membranes. In addition, as widely documented, rapid genetic manipulation in zebrafish is now routine, which coupled with the relative ease of cell and tissue imaging in translucent zebrafish embryos, makes this an ideal system for development and disease research. To further understand the contribution of autophagy during chondrocyte differentiation and maturation, and how this contributes to an accurate cartilage foundation for proper joint function, we used CRISPR-Cas9 to generate an *atg13* knockout zebrafish line [1]. Early *atg13^−/−^* zebrafish larvae (24 hpf) show no phenotypic differences from their wild-type or heterozygous counterparts, suggesting that global development is not overtly affected; however, by 5 dpf, phenotypes including short and/or bent body, edema, failure to utilize the yolk sac, and failure to inflate the swim bladder are apparent (the latter being consistent with recent evidence of autophagy-mediated surfactant secretion). In sum, these traits result in juvenile lethality at ~17 dpf. To determine which individual phenotypes might be the cause of lethality, we focused on joint development and function, based on video observations of restricted mouth and buccal movements in *atg13^−/−^* zebrafish larvae. Such disruptions to joint function pointed to a possible role for autophagy in supporting chondrocyte development, further highlighting how autophagy dysregulation may contribute to the development and progression of OA. Combined with evidence of inefficient yolk store nutrient mobilization, restricted mouth opening range in *atg13^−/−^* zebrafish at an age when they should be free-feeding would be likely to contribute to lethality. Indeed, similar outcomes have been recorded in *becn1/beclin1* and *atg7* null zebrafish, where defects in hepatic glycogen and lipid metabolism are linked to a failure to cope with metabolic stress at a similar stage.

Based on these observations, we applied a range of cell and tissue imaging approaches to decipher the likely causes of improper joint function in *atg13^−/−^* zebrafish, focusing on the zebrafish jaw which has a synovial joint and is a very good model of vertebrate joint development. Skeletal formation begins at ~2 dpf in zebrafish, with the establishment of craniofacial cartilaginous structures, so we compared cartilaginous templates of lower jaw elements in wild-type and *atg13^−/−^* zebrafish larvae by light and electron microscopy (EM). Our findings suggested that autophagy suppression accelerates chondrocyte maturation, leading to improper chondrocyte intercalation, with consequent disruptions to jaw joint formation and movement. EM images of chondrocytes at 5 dpf in *atg13^−/−^* fish revealed disrupted cellular organization, with autophagy-deficient chondrocytes showing evidence of premature hypertrophy associated with reduced vesicle exocytosis and a disordered surrounding extracellular matrix. Further evidence of advanced hypertrophy is seen in the expression of 2 key markers of chondrocyte maturation: Sox9a and Col10a1. In cartilage elements of the jaws of *atg13^−/−^* fish, Sox9a protein expression is reduced, with a premature switch to Col10a1 expression recorded accompanying reduced chondrocyte proliferation (Figure 1).

Given the established links between autophagy, cartilage health, and OA, our findings suggest several ways in which impaired autophagy can increase OA risk in patients, as aberrant chondrocyte maturation can: (i) alter joint functionality, leading to uneven joint loading throughout life; (ii) cause premature hypertrophy and untimely mineralization of articular cartilage; (iii) diminish matrix secretion increasing cartilage susceptibility to degeneration under normal physiological load. Further studies into how autophagy contributes to chondrocyte development, maturation, and maintenance could therefore contribute to strategies to decrease the risk of OA onset in an aging population.
